# The Relationship of Astrocytes and Microglia with Different Stages of Ischemic Stroke

**DOI:** 10.2174/1570159X21666230718104634

**Published:** 2023-09-25

**Authors:** Zhen Liang, Yingyue Lou, Yulei Hao, Hui Li, Jiachun Feng, Songyan Liu

**Affiliations:** 1 Department of Neurology, China-Japan Union Hospital, Jilin University, Changchun, China;; 2 Department of Rehabilitation, The Second Hospital of Jilin University, Changchun, China;; 3 Department of Neurology and Neuroscience Center, The First Hospital of Jilin University, Changchun, China

**Keywords:** Ischemic stroke, neuroinflammation, astrocytes, microglia, astrocyte-microglia interaction, stroke treatment

## Abstract

Ischemic stroke is the predominant cause of severe morbidity and mortality worldwide. Post-stroke neuroinflammation has recently received increasing attention with the aim of providing a new effective treatment strategy for ischemic stroke. Microglia and astrocytes are major components of the innate immune system of the central nervous system. They can be involved in all phases of ischemic stroke, from the early stage, contributing to the first wave of neuronal cell death, to the late stage involving phagocytosis and repair. In the early stage of ischemic stroke, a vicious cycle exists between the activation of microglia and astrocytes (through astrocytic connexin 43 hemichannels), aggravating neuroinflammatory injury post-stroke. However, in the late stage of ischemic stroke, repeatedly activated microglia can induce the formation of glial scars by triggering reactive astrogliosis in the peri-infarct regions, which may limit the movement of activated microglia in reverse and restrict the diffusion of inflammation to healthy brain tissues, alleviating the neuroinflammatory injury post-stroke. In this review, we elucidated the various roles of astrocytes and microglia and summarized their relationship with neuroinflammation. We also examined how astrocytes and microglia influence each other at different stages of ischemic stroke. Several potential therapeutic approaches targeting astrocytes and microglia in ischemic stroke have been reviewed. Understanding the details of astrocyte-microglia interaction processes will contribute to a better understanding of the mechanisms underlying ischemic stroke, contributing to the identification of new therapeutic interventions.

## 1 INTRODUCTION

Stroke is a multifactorial cerebrovascular disease with high morbidity and mortality, and ischemic stroke accounts for 75-80% of all strokes [1]. Depending on the size and location of the infarct region, stroke can cause varying degrees of cognitive, sensory, and/or motor impairment [2]. Currently, ischemic stroke, cardiovascular diseases, and malignant tumors are the three predominant causes of human death worldwide [3]. Effective treatments, such as intravenous thrombolysis with recombinant tissue plasminogen activator (rt-PA) and thrombectomy, have emerged in the past few years with the aim of restoring blood flow in ischemic regions within a narrow time window after the onset of ischemia [4]. Unfortunately, progressive neuronal degeneration and loss of function associated with treatment are difficult to avoid.

Accumulating evidence suggests that neuroinflammation plays a key role in I/R injury. Neuroinflammation could be beneficial by clearing dead cells and debris but may be detrimental if excessive, leading to cell dysfunction and cerebral edema [5]. The early stage of ischemic stroke (< 1 week) is characterized by damage, such as cytotoxic edema, cell death, neuroinflammation, extracellular matrix and tight junction protein degradation, and reperfusion injury. The late stage of ischemic stroke (> 1 week) is mainly characterized by repair, including neoangiogenic and recovery processes, neovascularization, and neurogenesis [6, 7].

Astrocytes and microglia are abundant in the central nervous system (CNS), where astrocytes account for 19-40% of glial cells and microglia constitute 5-20% of all glial cells [8]. Under normal physiological conditions, astrocytes and microglia can promote CNS homeostasis by providing structural and nutritional support to neurons [9]. In the adult brain, astrocytes and microglia are involved in neuromodulation, surveillance and monitoring, synaptic plasticity, learning, and memory. They are also responsible for maintaining blood-brain barrier (BBB) integrity and metabolic coupling, ion buffering, neurotransmitter homeostasis, production of neuroactive factors (ATP and TNF-α), and normal functioning circuits controlling neuronal synchronization and synapses [10]. In addition to homeostatic functions, under pathological conditions, astrocytes and microglia can respond to BBB disruption, energy failure, and inflammation induced by the reduction of cerebral blood flow after ischemic stroke [11-14]. Studies have shown that astrocytes could play dual roles after ischemic stroke, mainly by forming connexin 43 hemichannels or gap junctions [15]. After the onset of stroke, astrocytes can release neurotoxic factors *via* connexin 43 hemichannels to aggravate neuronal injury [16-18]. They also inhibit neuroinflammation and maintain CNS homeostasis with the astrocytic syncytium structure formed by connexin 43 gap junctions to rescue surviving neurons [19]. In addition, astrocytes are the central components of glial scars and although their role after ischemic stroke remains controversial [20, 21]. Some studies have also shown that astrocytes exhibit polarity changes following an ischemic stroke. The typical phenotypes of astrocytes, neurotoxic A1 phenotype, and neuroprotective A2 phenotype, play an essential biphasic function in ischemic stroke [22, 23]. A1 astrocytes (unhealthy astrocytes) highly upregulate several classical complement cascade genes to disrupt synapses. After stroke, activated microglia induce A1 astrocytes by secreting IL-1α, TNFα, and C1q [24]. A1 astrocytes lose the ability to promote neuronal survival, growth, synaptogenesis, and phagocytosis and induce the death of neurons and oligodendrocytes. In contrast, A2 astrocytes (healthy astrocytes) can be directly induced by ischemia, releasing many neurotrophic factors, strongly promoting neuronal survival and tissue repair, and regulating brain homeostasis [24-28]. However, the demarcation of A1/A2 polarities is now known to be oversimplified because astrocytes are considered to have multiple reactive phenotypes related to CNS [29].

Microglia are residential immune cells in the central nervous system that are in a quiescent state (M0 phenotype) under physiological conditions and play a role in immune surveillance. Under pathological conditions, microglia are rapidly activated and accompanied by adaptive transcriptional functional changes. Microglia have been divided into two different subtypes post-stroke: the M1/M2 phenotype [30]. Classically activated (M1 polarization) microglia release pro-inflammatory factors and toxic substances to kill pathogens. Microglia can recognize harmful stimuli and produce inflammatory cytokines (TNF-α, IL-6, IL-1 β, IFN γ, and multiple chemokines), which are essential for microglial polarization to the classical activated state “M1” [31]. Conversely, alternative activated (M2 polarization) microglia play a neuroprotective role by promoting tissue repair and regeneration [31]. There is no set description or classification for the M2 cells. The division of M2 macrophages is based on the observation that stimulation with various cytokines produces different receptor profiles, cytokine production, chemokine secretion, and function. M2 macrophages are characterized by the ability of their expressed mediators or receptors to downregulate, repair, or protect the body from inflammation [31]. However, with the increasing maturity of high-throughput single-cell techniques to access microglial heterogeneity, such as cytometry by time-of-flight mass spectrometry and single-cell RNA sequencing, an increasing number of researchers have found that microglia are activated into heterogeneous populations because the homogeneity of homeostatic microglia is readily disrupted under pathological conditions [32]. One type of heterogeneous microglia, disease-associated microglia (DAM), has received increasing attention. DAM presents a notable gene signature with high expression levels of Lpl (encoding lipoprotein lipase), Itgax (encoding cluster of differentiation (CD) 11c), Apoe, and Cst7 [33]. In response to disease progression, DAM leads to rapid changes in gene expression, resulting in a homeostatic type of microglia. DAM was detected primarily in disease-affected CNS regions but not in other regions. The DAM phenotype is a common feature of microglial responses to CNS pathology, independent of the disease etiology [34]. The discovery of DAM provides an opportunity to develop a therapeutic strategy that targets a common and intrinsic mechanism of neuronal death shared by multiple neurodegenerative diseases [34]. Disease-associated microglia include tumor-associated microglia (TAM) and non-tumor-associated microglia. TAMs in the tumor core are dominated by macrophages with anti-inflammatory M2-like properties, whereas the surrounding TAMs are dominated by microglia with more pro-inflammatory M1-like properties [35]. TAMs account for 30% of all tumors [35]. TAMs secrete various factors, such as cytokines and growth factors, that lead to or support different biological functions in the TME, such as stemness, proliferation, angiogenesis, cancer cell migration, and immunosuppression [35]. Nevertheless, the concept of phenotypic diversity is broadly accepted, although the simple dichotomized classification cannot reflect the complicated phenotypes of microglia [36, 37]. Therefore, the use of the M1/M2 nomenclature was limited in this review, appearing only directly from the references where it was used. For these reasons, microglia can be categorized into pro-inflammatory and anti-inflammatory phenotypes replacing the M1/M2 nomenclature after ischemic stroke [38]. Both pro- and anti-microglia are involved in regulating the function and status of neurons, astrocytes, and other glial cells [39].

Neuroinflammation mediated by astrocytes and microglia is involved in all stages of acute ischemic stroke, from the early stage, which leads to the first wave of neuronal cell death, to the late stages involving phagocytosis and tissue remodeling. In addition to the neuronal damage mentioned above, white matter injury caused by neuroinflammation after ischemic stroke has also attracted widespread attention [40, 41]. Wan *et al.* [42] reported that hypertrophic reactive astrocytes could phagocytose myelin and promote demyelination through the lipocalin-2/lipoprotein receptor-related protein 1 pathway after focal cortical ischemia in distal MCAO mice. Microglia-triggered inflammation also plays an important role in secondary white matter injury and subsequent white matter repair post-stroke [43, 44]. Pro-inflammatory microglia can secrete pro-inflammatory cytokines and exacerbate oligodendrocyte cell death and demyelination, thereby aggravating white matter injury [45]. In contrast, anti-inflammatory microglia can produce beneficial mediators and promote remyelination, thereby facilitating white matter repair [46]. In addition, cell-cell interactions between astrocytes and microglia can exist and play multifaceted roles in neuroinflammation after ischemic stroke. In the early stages of ischemic stroke, the vicious cycle between the activation of microglia and astrocytes through astrocytic connexin 43 hemichannels can aggravate neuroinflammatory injury post-stroke. In the late stage of ischemic stroke, repeatedly activated microglia can induce glial scar formation by triggering reactive astrogliosis in the peri-infarct regions, which may alleviate neuroinflammatory injury post-stroke. Therefore, in this review, we elucidated the roles of astrocytes and microglia, as well as the cell-cell interactions between them at different stages of ischemic stroke, with the aim of identifying potential targets for treatment of ischemic stroke.

## 2 ROLE OF ASTROCYTES AT DIFFERENT STAGES OF ISCHEMIC STROKE

Among the glial cells in the CNS, astrocytes are the most abundant, constituting almost 19-40% of glial cells, and may regulate homeostasis of the CNS environment and support the survival of neurons [21, 47]. Connexin 43 (Cx43), one of the most abundant connexins in the brain, is essential for astrocytes to perform their physiological functions by forming gap junctions and hemichannels [48, 49]. A number of astrocytes can form astrocytic syncytial structures to react to stimuli synchronously by forming Cx43/Cx43 gap junctions with other astrocytes [19]. Syncytium isoelectricity operates as a general mechanism in astrocyte networks [50]. Astrocytes in syncytial astrocytes are more like communities of destiny, whereas uncoupled astrocytes are not [50]. Under physiological and pathological conditions, astrocytic syncytium can protect astrocytes to a certain extent by sharing neuroprotective metabolites (including Na^+^, K^+^, glutamine (Glu), antioxidants, and glucose) and buffering toxic molecules (including Ca^2+^, excessive ATP, Glu, NO, and radical oxygen species (ROS)), further maintaining CNS homeostasis [51, 52]. Moreover, the astrocytic syncytium can also support the survival of neurons by maintaining ionic homeostasis [53], removing Glu in the synaptic cleft to avoid excitotoxicity [54] and providing energy substrates (glucose and lactate) to neurons [19]. Astrocyte Cx43 gap junctions play an essential role in astrocyte activation and removal of cytotoxic molecules, which promote neuronal survival [55]. In addition, the astrocyte network formed by gap junctions can provide energy substrates (glucose and lactate) to neurons [19]. Internalization of astrocytic Cx43 induced by C-terminal phosphorylation may contribute to astrocyte uncoupling, thereby reducing astrocyte mutual support and exacerbating post-ischemic stroke injury [56]. In ischemic stroke, hemichannel opening induced by reduced phosphorylation of Cx43 in astrocytes promotes the release of inflammatory mediators and increases neuroinflammation [57].

### 2.1 Astrocytes Promote Brain Injury in the Early Stage of Ischemic Stroke

After the onset of ischemic stroke, astrocyte function begins to change. For instance, astrocytes release Glu in the synaptic cleft, causing increased extracellular Glu [16]. The released Glu over activates Glu receptors and mediates excitotoxicity, eventually leading to neuronal death [58]. Glutamatergic stimulation results in elevated intracellular sodium and calcium levels, which leads to mitochondrial dysfunction, protease activation, reactive oxygen species accumulation, and nitric oxide release [58]. Two theories explain this phenomenon. One is that astrocytes can release Glu *via* astrocytic Cx43 hemichannels with increased permeability after ischemia [59]. The other is due to the reversible Glu transporters post-stroke, which means that Glu can be transported in the outward direction when extracellular (Na^+^) / intracellular (K^+^) decreases and/or intracellular (Na^+^)/ extracellular (K^+^) increases, which differs from the conventional inward direction [16].

Moreover, both *in vivo* and *in vitro* studies have demonstrated that increased astrocytic Ca^2+^ activity aggravates ischemic damage. In an *in vitro* study, we found that astrocytic Ca^2+^ activity is quiescent under normal conditions using Ca^2+^ imaging in brain slices, but frequent Ca^2+^ waves are detectable in oxygen-glucose deprivation (OGD) models. Furthermore, increased Ca^2+^ activity in the astrocytic syncytium was also found to play a key role in the activation of extrasynaptic NMDA receptors in hippocampal neurons, strengthening glutamatergic signaling and aggravating post-stroke brain damage. The same study also revealed that increased Ca^2+^ activity in the astrocytic syncytium plays a vital role in the activation of extrasynaptic NMDA receptors in hippocampal neurons, enhancing glutamatergic signaling, and brain damage post-stroke [17]. *In vivo* experiments in mice showed that astrocytic Ca^2+^ waves started 20 min after photothrombosis-induced ischemia, particularly in the ischemic core. Subsequently, the infarct volume was reduced when use BAPTA Ca^2+^ chelator to inhibit astrocytic Ca^2+^ activity [60].

Aquaporin 4 (AQP4) is a cellular membrane protein that contributes to water homeostasis in the CNS and is particularly expressed in astrocytic end-feet [61]. Recent studies have shown that astrocytes may be involved in the development of edema following ischemic stroke by increasing the expression of AQP4 [18]. Trifluoperazine (TFP) is a calmodulin antagonist that downregulates AQP4 membrane localization. By administering TFP to photothrombotic stroke mouse models, researchers confirmed that AQP4 mRNA and protein expression levels in the brain decreased, accompanied by significantly reduced cerebral edema [62]. In summary, in the early stage of post-stroke, astrocytes may reduce neuroinflammation, support neuron survival, and maintain CNS homeostasis with the astrocytic syncytium formed by gap junctions releasing Glu, although they may also enhance Ca^2+^ activity and upregulate AQP4 expression, which contributes to brain damage.

### 2.2 Astrocytes Inhibit Brain Injury in the Late Stage of Ischemic Stroke

In the late stage of stroke, astrocytes become hypertrophic and overexpress glial fibrillary acidic protein (GFAP). This proliferation process is the most characteristic feature of glial scars after stroke, and is called reactive astrogliosis. Several studies in mice using GFAP staining have observed that reactive astrogliosis started 4 days after MCAO [63]. In addition, glial scars formation was observed at 6 days post-stroke with scars becoming fully mature at 10 days post-stroke after photothrombosis-induced ischemia in adult mice [20]. Glial scar formation involves CNS cells and non-CNS cells including hematogenous macrophages and fibroblasts. Ischemic injury can cause direct death of neurons and glial cells in the ischemic core, releasing some factors associated with glial scar formation and immune response. Reactive astrocytes and microglia quickly react and migrate to the ischemic core, increasing the expression of pro-inflammatory factors and chemokines that inhibit axonal regeneration [20, 21]. Hematogenous macrophages also rapidly infiltrate the region attracting perivascular fibroblasts. Perivascular fibroblasts form the fibrotic part of the glial scar by releasing fibronectin and collagen type 1 into the core [64]. Within two weeks after stroke, the glial scar matures by forming tight borders between the fibrotic and glial components [65]. Previous studies have revealed that the role of glial scars depends on specific neuropathological conditions and cannot be simply defined as beneficial or detrimental. During ischemic stroke, glial scars can inhibit sprouting and axonal growth *via* pro-inflammatory factors (such as IL-6, TNF-α, IL-1α, and IL-1β) and free radicals (such as NO, superoxide, and peroxynitrite) [66]. In contrast, glial scars can play key roles in CNS repair and neurogenesis by producing or recycling neurotrophic factors [including brain-derived neurotrophic factor (BDNF) and neuronal growth factor (NGF) [67]. Glial scars can also separate the ischemic region from healthy tissue, preventing a cascading wave of uncontrolled tissue damage and restricting the diffusion of neuroinflammation [68]. Thus, in the late stage post-stroke, astrocytic morphology changes to hypertrophy and then forms glial scars with microglia, macrophages, and perivascular fibroblasts. Glial scarring plays a vital and complex role in acute ischemic stroke. The formation of glial scars after ischemic stroke may be determined by diverse molecules and signaling mechanisms. Therefore, future research should focus on these molecules and signal mechanisms, and how to modulate them to exert neuroprotective functions (Table **1**).

## 3 ROLE OF MICROGLIA AT DIFFERENT STAGE OF ISCHEMIC STROKE

Microglia are the resident immune cells of the CNS and make up almost 10% of the glial cells of the brain [8, 81]. Microglia behave similarly to peripheral macrophages, although they are derived from different sources: microglia are derived from myeloid progenitors, while peripheral macrophages are derived from hematopoietic stem cells [82, 83]. Under normal conditions, previous studies showed that astrocytes can secrete neurotransmitters (including ATP and Glu) [84] and neurons can express chemokines (including CD200 and CX3CL1) [85, 86] to bind to their receptors on microglia, maintaining microglia in a relatively quiescent state [87]. Microglia in the resting state have a small cell soma and several highly branched processes, monitoring damaged neurons and infectious agents of the CNS [88].

After an ischemic stroke, microglia become activated retracting their processes and displaying an amoeboid shape as well as rapid polarization into two different subtypes: the pro-inflammatory M1 phenotype or the anti-inflammatory M2 phenotype [30]. The M1 microglial phenotype can be characterized by surface markers such as CD16/32, CD86, CD40, and inducible nitric oxide synthase (iNOS) [89, 90]. CD206, a C-type lectin that functions in endocytosis and phagocytosis, is a classic marker of M2 microglia [90, 91]. Studies in animal stroke models revealed the polarization dynamics of microglia characterized by an increase in the M1 phenotype during the first 14 days in the peri-infarct region after ischemic stroke and an increase in the M2 phenotype in the first 5 days at the site of ischemic injury followed by a decline [45]. These data indicated that the classical activation of pro-inflammatory microglia was prolonged a long time after ischemic stroke, while anti-inflammatory microglia peaked in the early stage and then declined.

### 3.1 Pro-inflammatory M1 Phenotype Microglia Promote Brain Injury in Early and Late Ischemic Stroke

In the early stage post-stroke, M1 phenotype microglia are considered to cause damage to the surrounding neuronal cells by secreting pro-inflammatory factors (including IL-6, IL-1β, IFN-γ, TNF-α, IL-15, IL-18, and IL-23) [92, 93]. Several experiments on the neural factor CD200 have confirmed this. In the CNS, CD200 is primarily expressed by neurons, and its unique receptor, CD200R, is expressed by microglia. The CD200-CD200R interaction mainly plays a role in maintaining microglial cells in a quiescent homeostatic state. Experiments in transient middle cerebral artery occlusion (tMCAO) in rats revealed that CD200 in the ischemic core decreased rapidly compared to the contralesional healthy hemisphere in the first 48 h after ischemic stroke [94]. Another study in an ischemic mouse model reported that CD200 decreased after 48 h after permanent middle cerebral artery occlusion (pMCAO), contributing to microglial activation and associated neuronal death. However, intracerebroventricular injections of CD200 after induction of pMCAO reduced microglial activation and the expression of pro-inflammatory cytokines (including TNF-α and IL-1β), ameliorating neuroinflammation and associated neuronal death [95]. In the late stage of post-stroke, Sun *et al.* [96] found that the CD200-CD200R interaction could benefit spontaneous functional recovery (intrinsic recovery occurred after ischemic stroke) after 4-7 days in rats subjected to MCAO. Spontaneous functional recovery is achieved by inhibiting microglial activation and pro-inflammatory factor release, further enhancing synaptic plasticity. The researchers demonstrated that MCAO rats injected with CD200 showed better sensorimotor function, accompanied by enhanced synaptic plasticity, decreased microglial activation, and pro-inflammatory factor release. In contrast, intracerebroventricular injection of CD200R blocking antibody resulted in worse sensorimotor function, weakened synaptic plasticity, and increased microglia activation and release of pro-inflammatory factors. Altogether, pro-inflammatory microglia could aggravate neuroinflammation and neuronal death in the early stage and impede spontaneous functional recovery in the late stage after ischemic stroke. Inhibiting the levels of pro-inflammatory microglia is likely to have great efficacy. Further experiments are needed to verify this hypothesis.

### 3.2 Anti-inflammatory M2 Microglia Inhibit Brain Injury in the Early Stage of Ischemic Stroke

In both the early and late stages following ischemic stroke, anti-inflammatory M2 microglia are considered to be the major effector cell with the potential to counteract pro-inflammatory reactions by producing anti-inflammatory factors (IL-4, IL-10, IL-13, or transforming growth factor-β (TGF-β)) [30] as well as promote tissue repair and neurogenesis by producing insulin-like growth factor-1 (IGF1), BDNF, and NGF [97, 98]. Ischemic models in mice with tMCAO and pMCAO confirmed that M2 microglia-induced IL-4 secretion decreased infarct size and improved long-term functional recovery [99]. Zhu *et al.* [100] demonstrated that M2 microglia secrete chitinase-3-like protein 3 (Ym1/2), IL-10, and TGF-β to promote angiogenesis, decrease BBB leakage, and improve stroke outcomes in mice subjected to tMCAO. Additionally, experiments in tMCAO mice showed that M2 microglia upregulated TGF-α expression, thereby promoting the proliferation and neuronal differentiation of neural stem/progenitor cells in the ipsilateral subventricular zone [101]. Unfortunately, as mentioned above, anti-inflammatory microglia begin to decline soon after peaking in the early stages of ischemic stroke, which is detrimental to post-stroke functional recovery [45]. To promote CNS functional repair, further studies should focus on increasing the levels of anti-inflammatory microglia post-stroke.

## 4 INTERACTION BETWEEN ASTROCYTES AND MICROGLIA IN DIFFERENT STAGES OF ISCHEMIC STROKE

### 4.1 Early Stage of Ischemic Stroke

All astrocytes can communicate directly with neurons and other glial cells in the form of gap junctions. But they can also communicate indirectly with microglia through substances released from astrocytic Cx43 hemichannels, despite the fact that no gap junctions have been observed between astrocytes and microglia to date [102, 103]. Previous studies have demonstrated that the permeability of astrocytic Cx43 hemichannels is extremely low under physiological conditions [104]. After the onset of ischemic stroke, microglial activation can occur from minutes to a few hours after ischemic stroke [105, 106] and is driven by damage-associated molecular patterns (including high mobility group box 1, toll-like receptors [107] and peroxiredoxins [108]) from cell debris or apoptotic neuron cells [109]. Rupalla *et al.* observed microglial activation as early as 30 min after pMCAO in the penumbra using light microscopy [110]. Another study described the activation of microglia 24 h after pMCAO in cortical and thalamic regions by lectin histochemistry [111]. After activation, M1 microglia secrete pro-inflammatory factors, including tumor necrosis factor-alpha (TNF-α) and interleukin-1β(IL-1β) [112]. The hemichannels can then be activated by TNF-α and IL-1β resulting in increased permeability and enhanced release of ATP [113], which may activate the purine ionotropic receptors on microglia (including P2X4R and P2Y12R), thereby inducing more microglial polarization into the M1 subtype [114, 115]. In addition, activated astrocytic Cx43 hemichannels may also release Glu, which stimulated the microglial metabotropic glutamate receptor II, thus leading to the activation of M1 microglia through the NF-κB pathway [59, 116]. The M1 subtype can secrete pro-inflammatory factors, including TNF-α and IL-1β, aggravating neuroinflammation post-stroke [112]. *In vitro* studies have shown that TNF-α can induce apoptosis of neural stem cells [117]. Meme *et al.* co-cultured astrocytes with M1 subtype microglia induced by lipopolysaccharides *in vitro* and found that M1 subtype microglia inhibited astrocytic gap junctions and downregulated the expression of astrocytic Cx43 by secreting TNF-α and IL-1β [118, 119]. As mentioned above, astrocytes can form syncytial structures through gap junctions to jointly respond to ischemic stimuli as a whole. Thus, inhibition of gap junctions by the M1 subtype could uncouple astrocytes from astrocytic syncytial structures, weakening the tolerance response to ischemic stimuli of uncoupled astrocytes. Moreover, inhibition of Cx43 expression is considered to be correlated with enhanced activation of astrocytes and astrocytic proliferation, further forming a glial scar [120]. This process is described in detail below. TNF-α and IL-1β derived from activated microglia promote the activation of astrocytic Cx43 hemichannels, which in turn increase the release of ATP and Glu, further activating microglia [121, 122]. As a result, pro-inflammatory microglia-mediated neuroinflammation, abnormally opened astrocytic Cx43 hemichannels, and ATP and Glu release may complement each other, leading to a sequence of events that aggravates post-ischemic injury. However, similar interactions between astrocytes and anti-inflammatory microglia, which exert important neuroprotective effects after ischemia, have not yet been observed.

**Fig. (1):**
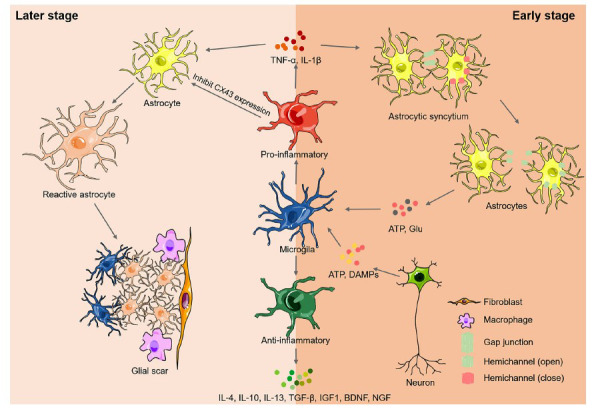
The interaction between microglia and astrocyte in different stages of ischemic stroke. After the onset of ischemic stroke, microglia retract their processes and display an amoeboid shape as well as rapid polarization to two different subtypes with the stimulation of ATP and damage-associated molecular patterns released mainly by neurons: the pro-inflammatory phenotype, or the anti-inflammatory phenotype. In the early stage of post-stroke, pro-inflammatory microglia may destruct gap junctions and increase the permeability of astrocytic Cx43 hemichannels by releasing TNF-α and IL-1β. Conversely, astrocytes may activate microglia to the pro-inflammatory phenotype through releasing ATP and Glu by opened astrocytic Cx43 hemichannels, enhancing injury after ischemic stroke. While in the late stage of post-stroke, repeatedly activated pro-inflammatory microglia could trigger reactive astrogliosis *via* releasing TNF-α and IL-1β as well as inhibiting the expression of astrocytic Cx43, further forming glial scar in the peri-infract region together with reactive astrocytes. In turn, the glial scar could incorporate pro-inflammatory microglia, thus limiting the movement of activated pro-inflammatory microglia. As a result, the glial scar separates the ischemic core from healthy brain tissue and restricts the diffusion of inflammation. In addition, it remains to be clarified whether in the early or late post-stroke stage, anti-inflammatory microglia could both exert anti-inflammatory functions by producing IL-4, IL-10, IL-13, or TGF-β, as well as promote tissue repair and neurogenesis by producing IGF1, BDNF, and NGF.

Therefore, in the early post-stroke stage, activated pro-inflammatory microglia could destroy gap junctions and increase the permeability of astrocytic Cx43 hemichannels by releasing pro-inflammatory factors. In contrast, astrocytes can activate microglia to the pro-inflammatory phenotype by releasing ATP and Glu *via* astrocytic Cx43 hemichannels, enhancing injury after ischemic stroke. This vicious cycle can cause significant damage to the brain tissue after ischemic stroke. Therefore, it is imperative to terminate the post-stroke cycle in future studies (Fig. **1**).

### 4.2 Late Stage of Ischemic Stroke

In the late stage of post-stroke, astrocytic morphological changes are associated with the upregulation of GFAP, which is called reactive astrogliosis [123]. It has been shown that neuroinflammation involves several inflammatory cytokines (such as TNF-α, IL-1β, IL-2, IL-6) which display vital roles in triggering reactive astrogliosis [64]. Studies in astrocytic Cx43-knockout mice also showed decreased expression of Cx43, as well as increased expression of GFAP and number of reactive astrocytes [120]. However, the exact molecular mechanism remains unclear as to whether the release of TNF-α and IL-1β or the downregulation of astrocytic Cx43 could be induced by M1 phenotype microglia, repeatedly activated in the cycle involving astrocytes and microglia in the early post-stroke stage discussed above. We assumed that repeatedly activated pro-inflammatory microglia release TNF-α and IL-1β as well as inhibit the expression of astrocytic Cx43 to a certain degree, in turn triggering significant reactive astrogliosis.

Wagner *et al.* further measured the dynamic modifications of reactive astrocytes after MCAO in spontaneously hypertensive rats using GFAP staining. The authors observed a thinner shape with elongated processes of reactive astrocytes in the penumbral region, compared with the astrocytes in the contralateral hemisphere and in remote regions away from the ischemic core 4 days after MCAO [63]. Li *et al.* observed the progression of glial scar formation by examining GFAP expression in a mouse model of photothrombotic ischemia at different time points after the induction of ischemia. In this study, they showed that the glial scar starts to form 6 days post-stroke and becomes completely mature 10 days post-stroke after photothrombosis-induced ischemia in adult mice [20]. These data showed that reactive astrogliosis, mainly in the peri-infarct region, could further form glial scars in the penumbra that separate the ischemic core from the healthy brain tissue.

Previous studies using histological and positron emission tomography technology have found that the distribution of activated microglia changes at different times post-stroke in humans. Most activated microglia are distributed in the ischemic core within 24-48 h after stroke onset and then mainly move to the periphery of the ischemic core at the late stage post-stroke, which may last for several weeks [124, 125]. Reactive astrocytes, along with microglia and macrophages, make up a large population of glial scars [64, 126]. As a result, the movement of activated pro-inflammatory microglia, which are distributed in the peri-infarct region, could be limited by the glial scar, thus restricting the diffusion of inflammation and benefiting spontaneous functional recovery at the late stage of post-stroke.

Therefore, in the late stage of post-stroke, repeatedly activated pro-inflammatory microglia could trigger reactive astrogliosis by releasing TNF-α and IL-1β as well as inhibiting the expression of astrocytic CX43, further forming glial scars in the peri-infarct region together with reactive astrocytes. In turn, the glial scar could limit the movement of activated pro-inflammatory microglia and separate the ischemic core from the healthy brain tissue to restrict the diffusion of inflammation, contributing to spontaneous functional recovery after ischemic stroke. Notably, glial scars can also play a detrimental role in inhibiting sprouting and axonal growth. Further research is required to determine whether glia scars have beneficial or detrimental effects (Fig. **1**).

## 5 THERAPIES AND APPLICATIONS

### 5.1 Potential Therapeutic Targets Act on Astrocytes for Ischemic Stroke

As the most abundant glial cells in the CNS, astrocytes play crucial supporting and regulatory roles in brain function and physiology. Depending on the timing and context, astrocytes may exacerbate inflammatory reactions and tissue damage or promote immunosuppression and tissue repair after ischemic stroke. Therefore, clarifying whether astrocytic reactivity and function should be reduced or further emphasized is clinically relevant. Several studies have reported that attenuated astrocyte responses often correlate with a decreased infarct size. Inhibiting nonspecific cyclin-dependent kinases could attenuate focal cerebral ischemia-induced astrocyte proliferation and delay neuronal death [127]. In addition, neuronal loss in the ischemic core 24 h after reperfusion and astrocyte proliferation in the boundary zone 14 days after ischemia were reduced by treatment with caffeic acid [128] or pranlukast, a cysteinyl leukotriene receptor 1 antagonist [129] in focal cerebral ischemic rats. However, it is difficult to determine the cause and effect due to differences in the time of neuronal loss and astrogliosis. Glu is the principal excitatory neurotransmitter in the CNS, and astrocytes may protect neurons from Glu excitotoxicity and oxidative stress after stroke *via* glutamate transporter-1 (GLT-1). Ceftriaxone, a GLT-1 transporter activator, could reduce neuronal injury in both hippocampal slice culture and ischemic rats through increasing GLT-1 expression in astrocytes [130, 131]. Tamoxifen, a selective estrogen receptor modulator, enhances the expression and function of GLT-1 in rat astrocytes [132]. Administration of tamoxifen reduces infarct volume and improves neurobehavioral outcomes after reversible MCAO in adult male rats [133] or pMCAO in adult female rats [134]. Additionally, carnosine [135], heat shock protein 72, and mitochondrial superoxide dismutase 2 [135] have been reported to exert neuroprotective effects by increasing GLT-1 expression in astrocytes in animal stroke models. Glu uptake by astrocytes is a critical mechanism for preventing excitotoxic neuronal death after stroke. It is still worth noting that the excess of Glu can also cause death of astrocytes. Both *in vitro* and *in vivo* studies have confirmed that FK506, an inhibitor of calcineurin, can efficiently inhibit glutamate-induced apoptosis of astrocytes early after reperfusion, thereby reducing excitotoxic neuronal death [136]. Experiments in rats following MCAO revealed that administration of TGF-α could reduce infarct size and improve functional recovery by promoting the sequential conversion of mature astrocytes into neural progenitors and stem cells [137, 138].

Other studies have demonstrated the therapeutic potential of increasing the expression of neurotrophic factors or decreasing the expression of neurotoxic factors in activated astrocytes to reduce ischemic injury. Galectin-1, a member of the family of beta-galactoside binding proteins, can induce astrocyte differentiation and strongly inhibit astrocyte proliferation, and differentiated astrocytes greatly enhance their production of BDNF [139]. Administration of galectin-1 to rats subjected to photochemical ischemic stroke enhanced the expression and secretion of astrocytic BDNF, reduced neuronal apoptosis in the ischemic boundary zone, and improved functional recovery [140]. Pyruvate, the end product of glycolysis, was found to stabilize hypoxia-inducible factor-1α in both neurons and astrocytes, thereby driving endogenous pyruvate erythropoietin synthesis and protecting the brain against ischemia-reperfusion injury [141]. In addition, astrocytes have been shown to produce monocyte chemoattractant protein-1 (MCP-1) in the rat brain following focal cerebral ischemia. Additionally, intracerebroventricular injection of viral macrophage inflammatory protein-II, a broad-spectrum chemokine receptor antagonist, reduced infarct volume in a dose-dependent manner [142]. These findings indicate that MCP-1 produced by astrocytes plays a detrimental role in ischemic injury and that its receptors are potential targets for therapeutic intervention in stroke. In recent years, the detrimental role of astrocytic connexin 43 hemichannels has attracted increasing attention. Several astrocytic Cx43 targeted agents have been considered as potential therapeutic agents for ischemic stroke. Cx43 mimetic peptides (including gap 19, gap 26, gap 27, peptide 5, and L2 peptide) [143, 144], carbenoxolone [145] and leptin [146] can serve as connexin 43 hemichannel blockers, further reducing ischemic injury in animal stroke models.

### 5.2 Anti-inflammatory M2 Microglia Inhibit Brain Injury in the Early Stage of Ischemic Stroke

**Fig. (2):**
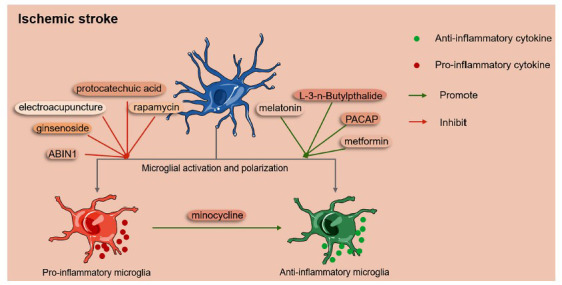
Drugs targeting the activation and polarization of microglia. After the onset of ischemic stroke, microglia retract their processes and display an amoeboid shape as well as polarization to two different subtypes rapidly: the pro-inflammatory phenotype, or the anti-inflammatory phenotype. Pro-inflammatory microglia could release pro-inflammatory cytokines such as IL-6, IL-1β, IFN-ϒ, TNF-α, IL-15, IL-18, IL-23 [80, 81], aggravating the neuroinflammatory injury post-stroke. Anti-inflammatory microglia could release anti-inflammatory cytokines like IL-4, IL-10, IL-13, TGF-β [30] thereby attenuating the post-stroke neuroinflammatory injury. Different drugs are used as neuroprotective agents to treat ischemic stroke by acting on these events. Ginsenoside, protocatechuic acid, rapamycin, ABIN1 and electroacupuncture therapy can inhibit the microglial switch to pro-inflammatory phenotype, thus inhibiting the release of pro-inflammatory cytokines. Metformin, melatonin, L-3-n-Butylpthalide, and PACAP can promote the microglial switch to anti-inflammatory phenotype. In addition, minocycline can switch pro-inflammatory microglia to anti-inflammatory microglia, further promoting the release of anti-inflammatory cytokines.

As the resident immune cells of the CNS, microglia undergo morphological changes from a ramified to an amoeboid state after ischemic stroke [147, 148]. They are then activated and polarized into two different phenotypes: the pro-inflammatory M1 phenotype and the anti-inflammatory M2 phenotype [44, 83, 149]. Accumulating evidence has shown that the ratio of the M1 and M2 phenotypes determines the neuroinflammatory fate post-stroke, contributing to varying degrees of ischemic injury [150, 151]. Current research has tested various agents that act on the polarization of microglia by decreasing pro-inflammatory microglia or increasing anti-inflammatory microglia, and have acquired neuroprotective efficacy in the treatment of ischemia (Fig. **2**).

To date, therapeutic agents aimed at decreasing pro-inflammatory microglia mainly include ginsenoside, protocatechuic acid, rapamycin, A20-binding inhibitor of NF-κB 1 (ABIN1), and electroacupuncture therapy. Ginsenoside treatment has been shown to improve neurological outcomes in patients suffering from ischemic stroke in clinical trials by inhibiting microglial activation and decreasing pro-inflammatory cytokine production related to M1 microglia [44]. Protocatechuic acid could suppress the activation of M1 microglia in mice with intracerebral hemorrhage, which may play similar roles in ischemic stroke [152]. The mTOR inhibitor rapamycin could reduce the Iba1^+^ cell count and the expression of pro-inflammatory markers correlated with M1 microglia, reducing infarct size in rats [152, 153]. ABIN1, an NF-κB inhibitor, ameliorates neuroinflammation by inhibiting M1 microglia in cerebral ischemia/reperfusion rats [154]. Along with these agents, electroacupuncture therapy can also decrease M1 microglia by inhibiting NF-κB signaling, and it also prevents the expression of Myd88 and P38-MAPK (P38-mitogen-activated protein kinase), which are involved in the inflammatory cascade in cerebral ischemic rats [154-156].

Abundant studies have also explored drugs such as metformin, melatonin, L-3-n-Butylpthalide, and pituitary adenylate cyclase-activating polypeptide (PACAP), aiming to increase the number of anti-inflammatory microglia that release anti-inflammatory cytokines, thereby attenuating the post-stroke inflammatory response. AMPK is known that AMPK is a major factor contributing to the induction of microglia/macrophage polarization toward the M2 phenotype in the ischemic brain, producing anti-inflammatory roles [157]. Metformin is generally used as a therapeutic drug for type 2 diabetes but has been proven to increase microglial M2 polarization by activating AMPK *in vivo* and *in vitro*, thus promoting angiogenesis [44]. Melatonin can reduce infarct size by promoting M2 polarization through the signal transducer and activator of transcription (STAT) 3 pathway in mice with MCAO [158, 159] and inhibits the release of IL-1β in a cellular ischemic model induced by oxygen-glucose deprivation [160]. Studies have shown that L-3-n-butylphthalide (extract of Apium graveolens seeds) increases M2 microglia after administration for one week in mice following MCAO [161]. PACAP is a 38-amino acid neuroprotective peptide that was initially isolated from hypothalamus extracts [162]. A study found that delivering PACAP into mouse brains three days after electrocauterization of the right middle cerebral artery can cause microglial polarization to the M2 phenotype on 7-14 day, thus enhancing functional recovery [163].

Minocycline has also been shown to facilitate microglial polarization from the M1 phenotype to M2 through the STAT1/6 pathway, thus alleviating inflammation and cell death after ischemic stroke [164]. An open-label, evaluator-blinded study demonstrated that the modified Rankin score (mRs) of patients suffering from ischemic stroke can decrease if minocycline is administered within 6 to 24 h following symptom onset [165]. However, another multicenter randomized, double-blind, placebo-controlled trial showed that there are no long-term beneficial effects on neurological outcomes between administration of either minocycline or placebo within 3-48 h after symptom onset [166]. Therefore, the actual efficacy of minocycline requires further expansion of the sample size for confirmation.

### 5.3 Potential Therapeutic Agents Act on Astrocytes and Microglia for Ischemic Stroke

Interestingly, some potential agents can affect both astrocytes and microglia. In a model of rat MCAO, previous studies found that cottonseed oil treatment could alleviate ischemic stroke injury by reducing microglial and astrocytic activation and inflammation, which was related to the inhibition of Toll-like receptor 4 (TLR4)/NF-κB pathway and the reduction of neurotoxic A1 phenotype astrocyte activation [167]. In addition, treatment with IL-11 exerted a neuroprotective effect by inhibiting the ischemia-induced activation of astrocytes and microglia in a model of focal cerebral ischemia [168]. Given the interaction between astrocytes and microglia, some agents may act directly on one and then indirectly on the other. For example, a clinical study of 259 ischemic stroke patients demonstrated a positive correlation between higher interleukin‐33 (IL-33) levels and a better prognosis [169]. When the CNS is injured, IL-33 is upregulated and exhibits a nuclear localization in astrocytes [170]. To examine the neuroprotective mechanism of IL-33, studies in a tMCAO model revealed that the expression level of IL‐33 rapidly increases in astrocytes after stroke. Similarly, the receptor of IL-33 (ST2) was highly expressed in microglia (according to flow cytometry analyses) and increased rapidly after stroke. In the tMCAO model, an ST2 deficiency in mice exacerbated brain infarction and neurological deficits; intracerebroventricular infusions of IL‐33 attenuated brain infarction [171]. These results strongly suggest that IL‐33/ST2 signaling is a beneficial interaction between astrocytes and microglia. Moreover, further *in vitro* studies on co‐culture systems have demonstrated that IL‐33/ST2 signaling can potentiate the expression of M2‐related genes such as IL‐10 in primary microglia, which are critical to obtain neuroprotective effects [172]. Based on this neuroprotection from activated IL-33 receptors expressed by microglia, agents that can promote the expression and rapid release of IL-33 by astrocytes may be promising for the prevention and treatment of stroke.

## CONCLUSION

Astrocytes and microglia exert complex regulatory effects in response to ischemia-induced neuroinflammation. An in-depth understanding of their various roles at different stages of ischemic stroke can help us understand the pathology of ischemic stroke. In this study, we demonstrated that astrocytes can damage brain tissue by releasing Glu, enhancing Ca^2+^ activity, and upregulating AQP4 expression; however, they can also protect brain tissue by their astrocytic syncytium structure formed by gap junctions in the early stage of ischemic stroke. In the late stage of ischemic stroke, astrocytes can form glial scars with other cells (including microglia, macrophages, and perivascular fibroblasts). Glial scars can inhibit sprouting and axonal growth by pro-inflammatory factors (IL-6, TNF-α, IL-1α, and IL-1β) and free radicals (NO, superoxide, and peroxynitrite), or promote CNS repair by neurotrophic factors (BDNF and NGF). In addition, glial scars can separate the ischemic region from healthy tissue, restricting the diffusion of neuroinflammation. After the onset of ischemic stroke, microglia are activated and polarize into pro-inflammatory or anti-inflammatory phenotypes. Pro-inflammatory microglia generally play detrimental roles by secreting pro-inflammatory factors, which aggravate neuroinflammation and neuronal death in the early stages and impede spontaneous functional recovery in the late stages after ischemic stroke. In contrast, anti-inflammatory microglia exert beneficial effects by releasing anti-inflammatory cytokines and neurotrophic factors, which promote phagocytosis and repair during all post-stroke phases. Further research is necessary to determine how to effectively apply these mechanisms in future stroke treatment.

More importantly, the cell-cell interaction between astrocytes and microglia post-stroke can produce a wider range of effects. Studies on these crosstalk pathways will help to understand the underlying pathological mechanisms of ischemic stroke. In the early stage post-stroke, pro-inflammatory microglia can induce the destruction of gap junctions and increase the permeability of hemichannels by secreting TNF-α and IL-1β. Subsequently, astrocytic Cx43 hemichannels are abnormally activated and release ATP and Glu, activating microglia to the pro-inflammatory phenotype in reverse, aggravating injury after ischemic stroke. Subsequently, in the late post-stroke stage, repeatedly activated pro-inflammatory microglia from the cycle involving the activation of microglia and astrocytes could trigger reactive astrogliosis, further forming a glial scar in the peri-infarct region. As a result, glial scars could limit the movement of activated microglia and separate the ischemic core from healthy brain tissue to re-establish CNS homeostasis, contributing to spontaneous functional recovery after ischemic stroke. However, it cannot be ignored that glial scars could also inhibit sprouting and axonal growth, which is detrimental for recovery after ischemic stroke. Overall, the interaction between astrocytes and microglia plays a dual role after an ischemic stroke. In the early stage of ischemia, astrocyte-microglia interaction amplifies the inflammatory cascade and exacerbates ischemic injury. In the late stage, although the diffusion of inflammation is limited by the formation of a glial scar, it also hinders axonal growth and sprouting. Therefore, further research should focus on selecting a key point in the interaction as a target for intervention to regulate the inflammatory response after ischemia.

Based on the interaction of astrocytes and microglia, pro-inflammatory microglia are detrimental and crucial in both the early and late stages of post-stroke. However, anti-inflammatory microglia always display beneficial effects in all post-stroke phases. The ratio of pro-inflammatory to anti-inflammatory microglia may determine the degree of ischemic injury. To date research has shown the neuroprotective effects of several therapeutic agents in ischemic stroke by reducing pro-inflammatory microglia, increasing anti-inflammatory microglia, or promoting the conversion of pro-inflammatory microglia to anti-inflammatory microglia *in vivo* and *in vitro*. Therefore, reducing pro-inflammatory microglia, increasing anti-inflammatory microglia, or promoting the conversion of pro-inflammatory microglia to anti-inflammatory microglia are potentially effective strategies in ischemic stroke. Future research should build on these theoretical foundations to explore safer and more efficient drugs. In addition, as the most abundant glial cells in the CNS, astrocytes play detrimental or beneficial roles after ischemic stroke, depending on timing and location, indicating that astrocytes are a promising therapeutic target. Therefore, to develop successful clinically relevant neuroprotective and neurorestorative strategies, additional research on astrocytes is needed.

## Figures and Tables

**Table 1:** Different effects of signal pathways on glial scar formation and ischemic stroke.

**Signal Pathways**	**Glial Scar Formation**	**Ischemic Stroke**
GFAP	Promote [69]	Beneficial [70, 71]
P38-MAPK	Inhibit [72]	Detrimental [73]
STAT3	Promote [74, 75]	Detrimental [76]
TGF-β	Promote [77, 78]	Beneficial [79]
CD36	Promote [80]	Detrimental [80]

**Abbreviations**: GFAP = Glial fibrillary acidic protein; P38-MAPK = P38-mitogen-activated protein kinase; STAT3 = Signal transducer and activator of transcription 3; TGF-β = Transforming growth factor-β; CD36 = Cluster of differentiation.

## LIST OF ABBREVIATIONS

ABIN1: A20-binding Inhibitor of NF-κB 1
AQP4: Aquaporin 4
BBB: Blood Brain Barrier
BDNF: Brain-derived Neurotrophic Factor
CD: Cluster of Differentiation
CNS: Central Nervous System
Cx43: Connexin 43
DAM: Disease-associated Microglial
GFAP: Glial Fibrillary Acidic Protein
GLT-1: Glutamate Transporter-1
Glu: Glutamine
IGF-1: Insulin-like Growth Factor-1
iNOS: Inducible Nitric Oxide Synthase
MCP-1: Monocyte Chemoattractant Protein-1
mRs: Modified Rankin Score
NGF: Neuronal Growth Factor
OGD: Oxygen-glucose Deprivation
P38-MAPK: P38-mitogen-activated Protein Kinase
PACAP: Pituitary Adenylate Cyclase-activating Polypeptide
pMCAO: Permanent Middle Cerebral Artery Occlusion
ROS: Radical Oxygen Species
rt-PA: Recombinant Tissue Plasminogen Activator
STAT: Signal Transducer and Activator of Transcription
TFP: Trifluoperazine
TGF-β: Transforming Growth Factor-β
tMCAO: Transient Middle Cerebral Artery Occlusion
TNF-α: Tumor Necrosis Factor-alpha
TLR4: Toll-like Receptor 4
